# Increase of Parkin and ATG5 plasmatic levels following perinatal hypoxic-ischemic encephalopathy

**DOI:** 10.1038/s41598-022-11870-w

**Published:** 2022-05-12

**Authors:** Anna Tarocco, Giampaolo Morciano, Mariasole Perrone, Claudia Cafolla, Cristina Ferrè, Tiziana Vacca, Ginevra Pistocchi, Fabio Meneghin, Ilaria Cocchi, Gianluca Lista, Irene Cetin, Pantaleo Greco, Giampaolo Garani, Marcello Stella, Miria Natile, Gina Ancora, Immacolata Savarese, Francesca Campi, Iliana Bersani, Andrea Dotta, Eloisa Tiberi, Giovanni Vento, Elisabetta Chiodin, Alex Staffler, Eugenia Maranella, Sandra Di Fabio, Mariusz R. Wieckowski, Carlotta Giorgi, Paolo Pinton

**Affiliations:** 1 Neonatal Intensive Care Unit and Neonatology, University Hospital S. Anna, 44121 Ferrara, Italy; 2 Department of Medical Sciences, Laboratory for Technologies of Advanced Therapies (LTTA), University of Ferrara, Via Fossato di Mortara 70, 44121 Ferrara, Italy; 3 Department of Medical Sciences, Pediatric Unit, University of Ferrara, 44121 Ferrara, Italy; 4 BSC Medical Biosciences Faculty of Medicine, Imperial College, London, SW7 2AZ UK; 5 Neonatal Pathology and Neonatal Intensive Care Unit, Vittore-Buzzi Children Hospital, Milan, Italy; 6 Obstetrics and Gynecology Unit, Vittore Buzzi Children Hospital” and University of Milan, 20154 Milan, Italy; 7 Department of Medical Sciences, Section of Obstetrics and Gynecology, University Hospital S.Anna, 44121 Ferrara, Italy; 8 Pediatrics Department and Neonatal and Pediatric Intensive Care Unit, Bufalini Hospital, 47521 Cesena, Italy; 9 Neonatal Intensive Care Unit, Infermi Hospital Rimini, 47921 Rimini, Italy; 10 Department of Neonatology, Bambino Gesù Children’s Hospital - IRCCS, 00165 Rome, Italy; 11 Department of Woman and Child Health, Obstetric and Neonatology Area, Fondazione Policlinico Universitario A. Gemelli IRCCS, 00168 Rome, Italy; 12 Division of Neonatology, Central Teaching Hospital of Bolzano, 39100 Bolzano, Italy; 13 Neonatology and Neonatal Intensive Care Unit, San Salvatore Hospital, 67100 L’Aquila, Italy; 14 Laboratory of Mitochondrial Biology and Metabolism, NenckiInstituteofExperimental Biology, Polish Academy of Sciences, 02-093 Warsaw, Poland

**Keywords:** Diseases, Neurology, Pathogenesis

## Abstract

Brain injury at birth is an important cause of neurological and behavioral disorders. Hypoxic-ischemic encephalopathy (HIE) is a critical cerebral event occurring acutely or chronically at birth with high mortality and morbidity in newborns. Therapeutic strategies for the prevention of brain damage are still unknown, and the only medical intervention for newborns with moderate-to-severe HIE is therapeutic hypothermia (TH). Although the neurological outcome depends on the severity of the initial insult, emerging evidence suggests that infants with mild HIE who are not treated with TH have an increased risk for neurodevelopmental impairment; in the current clinical setting, there are no specific or validated biomarkers that can be used to both correlate the severity of the hypoxic insult at birth and monitor the trend in the insult over time. The aim of this work was to examine the presence of autophagic and mitophagic proteins in bodily fluids, to increase knowledge of what, early at birth, can inform therapeutic strategies in the first hours of life. This is a prospective multicentric study carried out from April 2019 to April 2020 in eight third-level neonatal intensive care units. All participants have been subjected to the plasma levels quantification of both Parkin (a protein involved in mitophagy) and ATG5 (involved in autophagy). These findings show that Parkin and ATG5 levels are related to hypoxic-ischemic insult and are reliable also at birth. These observations suggest a great potential diagnostic value for Parkin evaluation in the first 6 h of life.

## Introduction

Perinatal asphyxia refers to a condition in which impaired gas exchange leads to fetal hypoxemia and hypercarbia. Its frequency is approximately 2–3/1000 live births^1^. Hypoxic-ischemic encephalopathy (HIE) is considered a multifactorial phenomenon in which several hypoxic, inflammatory, and traumatic events affect the fetal/neonatal brain^2,3^. Left untreated, up to 50% of asphyxiated newborns die, while 25% of survivors develop permanent neurological disabilities^4^. To identify affected infants, the evaluation of maternal history, obstetrical antecedents, intrapartum factors and placental pathology are necessary. HIE can develop acutely or chronically during the prenatal (hypoxia), perinatal (umbilical cord accidents, placental factors) or postnatal period (cardiac arrest)^5^. HIE at birth is diagnosed with a combination of laboratory hematic analyses (metabolic acidosis) or perinatal events (resuscitation (CPR), low Apgar score), neurological signs (graded as mild-moderate and severe using the clinical Sarnat Grading System) and neuromonitoring tool (aEEG or conventional EEG)^6,7^. To tailor a treatment in the clinical setting, however, it is extremely important to identify the origin and timing of the damaging event. The only medical intervention able to reduce mortality and morbidity in HIE is therapeutic hypothermia (TH). However, neurodevelopmental benefits are observed only when TH is initiated within the first 6 h of life^8^ and only in select patients who meet the specific criteria for TH. One should also consider that although the neurological outcome depends on the severity of the initial insult, infants with mild HIE who are not treated with TH have an increased risk for neurodevelopmental impairment^4,9^. In principle, these infants are considered to have a good prognosis; nevertheless, the neurological outcome of mild HIE beyond the newborn period has not been assessed, and few randomized controlled trials with TH have been drawn for those babies. Considering the high relevance of this condition, the lack of effective therapeutic approaches, and the difficulties in the prediction, detection, and grading of neonatal HIE, a better understanding of the pathological mechanisms accompanying it and the identification of reliable diagnostic and prognostic biomarkers would increase our knowledge in applying neuroprotective strategies. Numerous blood biomarkers for the evaluation of perinatal HIE have been studied^10–12^; however, none appears to help in the early identification of babies at risk for neurological impairment to guide neuroprotective strategies^13^. Glial fibrillary acidic protein (GFAP), for example, has already been correlated with the damage detected with MRI; however, its variation in the serum is not statistically relevant during the first 6 h of life and cannot be used to guide therapeutic decisions that must be made in the therapeutic window. Likewise, neuron-specific enolase (NSE), MBP, S100B and Tau increase following brain damage, but they either have not been studied at birth or their levels start to be significantly higher only after a few hours. Others, such as UCHL-1, rapidly decrease, making the assessment of damage impractical over time. In neonatal HIE, neuronal injury is mainly the result of apoptotic and necrotic death. However, increased expression of autophagy and mitophagy markers has been detected in postmortem samples of the basal ganglia, thalamus, cortex, and hippocampus of asphyxiated newborns^14–17^.

Autophagy allows the removal of unnecessary and dysfunctional components or their recycling to sustain energy requirements. In particular, Autophagy related 5 (ATG5) is essential in both canonical and noncanonical autophagy^18,19^, and its depletion significantly downregulates autophagy activation. In the case of mitochondria, autophagy is referred to as mitophagy and is responsible for the removal of mitochondria tagged as dysfunctional. One critical protein, namely, Parkin, marks mitochondria that are ready to be digested^20,21^. Previously published studies found ATG5 and Parkin to be easily detectable in body fluids. Interestingly, these proteins are considered diagnostic tools for the early monitoring of patients with cognitive decline^22^ and for identifying the active phase of multiple sclerosis^23^. This work aims to gain clinical insights linking autophagy and mitophagy modulation at birth and during the first days of life to investigate the correlation between the severity of HIE and the plasma levels of autophagic and mitophagic proteins.

## Methods

### Study population

This is a prospective multicentric study carried out from April 2019 to April 2020 in Italian eight third-level neonatal intensive care units, the first being located in Ferrara, where the study is promoted. It was approved by the local Ethics Committee and recorded to Clinical Trial as AutophaGy AcIdosis Newborn – AGAIN” (NCT03897101). The study cohort comprised infants, inborn or outborn, born after 35 weeks’ gestational age (GA), having a birth weight greater than 1800 gr, age ≤ 6 h, and written parental informed consent.

Newborns were divided into 3 groups following the Italian Guidelines for Hypotermic treatment for hypoxic-ischemic encephalopathy^34^.

Group A: newborns who meet all the criteria for the hypothermic treatment.

Criterion A) Intrapartum hypoxia data defined by at least one of the following criteria:

- pH of ≤ 7.0 or a base deficit of ≥ 12 mmol/L in a sample of umbilical cord blood or arterial analysis within 1 h of life or.

- Apgar scores less than five at ten minutes;

- the requirement for positive pressure ventilation, at 10 min.

Criterion B) Moderate or severe hypoxic-ischemic encephalopathy assessed between 30 and 60 min of life according to the neurological examination by Sarnat.

Criterion C) Pathological aEEG/EEG.

Are also eligible to TH all the neonates with mild hypoxic-ischemic encephalopathy and pathological aEEG/EEG.

Group B: newborns who not meet all the criteria for the hypothermic treatment with the following characteristics.

Criterion A) Intrapartum hypoxia data defined by at least one of the following criteria:

- pH of ≤ 7.0 or a base deficit of ≥ 12 mmol/L in a sample of umbilical cord blood or arterial analysis within 1 h of life or

- Apgar scores less than five at ten minutes;

-The requirement for positive pressure ventilation, at 10 min.

Criterion B) mild encephalopathy or normal neurological examination assessed between 30 and 60 min of life according to the neurological examination by Sarnat&Sarnat.

Criterion C) normal aEEG/EEG tracing.

This population is not being eligible for TH according to the Italian Guidelines.

Group C: control group (healthy newborns).

Obstetric, perinatal and neonatal clinical data were recorded in a specific Excel database.

According to the literature, the Sarnat score evaluated six categories: level of consciousness, spontaneous activity, posture, tone, primitive reflexes (suck and Moro), and autonomic nervous system (pupils, heart rate, and respiration). Each category was scored for pre-defined signs consistent with normal, mild, moderate, or severe. Infants with ⩾ 1 abnormal category but no evidence of moderate or severe neonatal encephalopathy (defined as moderate and/or severe abnormality in three categories) were classified as mild neonatal encephalopathy^24^.

### Autophagy and mitophagy detection and quantification

Five hundred microliters of blood samples were collected from the arteries or heels of neonates and centrifuged at 1000 rpm for 15 min at 4 °C to obtain plasma. Plasma at T0 (at birth), T1 (between 48 and 72 h after birth, with metabolic screening mandatory by law) and, for groups A and B, at T2 (7 days after birth) was used for autophagy and mitophagy quantification via commercially available enzyme-linked immunosorbent assays (ELISA). The ELISAs were used to determine circulating levels of proteins involved in the autophagy pathway, such as ATG5 (MyBiosource, MS7209535), and in mitophagy, such as Parkin (MyBiosource, MBS732278), with high sensitivity (ATG5: 0.1 ng/ml; Parkin: 1 pg/ml), a high detection range (ATG5: 1–100 ng/ml; Parkin: 1 – 5000 pg/ml) and excellent specificity with no significant cross-reactivity among protein analogs.

### Data analysis

Statistical analyses were performed by using GraphPad Prism. To analyze the differences among experimental groups (healthy control-group C, not needing TH cohort B and severe asphyxia-group A), one-way ANOVA was carried out. If any, outliers were removed by using the ROUT method with Q = 1% in GraphPad Prism.

## Results

This study was conducted on 243 newborns divided into the following clinical categories: 83 babies in the B group (not needing TH) and 57 with severe asphyxia who received TH (group A). Both were compared with 103 age-matched healthy newborns (group C) whose blood samples were collected at different time points: T0 (at birth), T1 and, for group A and B at T2.

The clinical features of the whole cohort are reported in Table 1 and the maternal and perinatal characteristics of the study population are reported in Table 2.

**Table 1:** General characteristics of the studied population.

Infant characteristics	Group A N = 57	Group B N = 83	Group C N = 103
Gestational age weeks (mean ± sd)	39.6 ± 1.79	39.76 ± 1.34	39.61 ± 1.32
Birth weight gr (mean ± sd)	3184 ± 569	3320.43 ± 483.29	3367.23 ± 427.39
Male, n (%)	31 (54.3%)	46 (55.42%)	59 (57.28%)
Female, n (%)	26 (45.6%)	37 (44.58%)	44 (42.72%)
*Resuscitation at birth, n (%)*	39 (68.4%)	57 (68.67%)	8 (7.77%)
CPAP	5 (8.7%)	31 (37.35%)	4 (3.88%)
PPV	19 (33.3%)	25 (30.12%)	4 (3.88%)
Endotracheal intubation	10 (17.5%)	0	0
Chest compression	5 (8.7%)	1 (1.2%)	0
Apgar score ≤ 5 at 1’ n (%)	40 (70%)	34 (40.96%)	5 (4.85%)
Apgar score ≤ 5 at 5’ n (%)	21 (36.8%)	3 (3.61%)	0
*Blood gas*			
Arterial pH (mean ± sd)	7.04 ± 0.15	7.04 ± 0.11	7.27 ± 0.08
Arterial base deficit (mean ± sd)	− 16.3 ± 5.0	− 15.1 ± 2.4	− 7.02 ± 3.62
Lactate mmol/L (mean ± sd)	11.0 ± 4.11	11.4 ± 4.5	5.42 ± 2.9

GBS, Streptococcus Agalactiae; PROM, premature rupture of membranes; CS, cesarean section; CPAP, continuous positive airway pressure; PPV, positive pressure ventilation.

**Table 2:** Maternal and perinatal characteristics of the study population.

Maternal characteristics	Group A	Group B	Group C
Gestational hypertension	3 (5.2%)	6 (7.23%)	4 (3.88%)
Diabetes mellitus	1 (1.7%)		
Gestational diabetes (diet/insulin)	5 (8.7%)	17 (20.48%)	12 (11.65%)
*Perinatal characteristics*			
Fever	5 (8.7%)	6 (7.23%)	2 (1.94%)
GBS +	6 (10.5%)	16 (19.28%)	16 (15.53%)
PROM > 18 h	9 (15.7%)	9 (10.84%)	9 (8.74%)
Intrapartum antibiotic	5 (8.7%)	15 (18.07%)	12 (11.65%)
prophylaxis	Not completed	Not completed	Not completed
	4 (7%) Completed	1 (1.2%) Completed	4 (3.88%) Completed
Cardiotocography abnormalities n (%)	22 (38.5%)	23 (27.71%)	3 (2.91%)
Sentinel events n (%)	12 (21%)	25 (30.12%)	0 (%)
Meconium-stained amniotic fluid, n (%)	20 (35%)	27 (32.53%)	25 (24.27%)
Normal spontaneous vaginal delivery	18 (31.6%)	36 (43.37%)	40 (38.83%)
Vaginal delivery with instruments	9 (15.7%)	11 (13.25%)	3 (2.91%)
Cesarean section	3 (5.2%)	6 (7.23%)	20 (19.42%)
Emerging CS, n (%)	18 (31.5%)	12 (14.46%)	12 (11.65%)

### Asphyxiated neonates have the highest levels of Parkin

**Figure 1:**
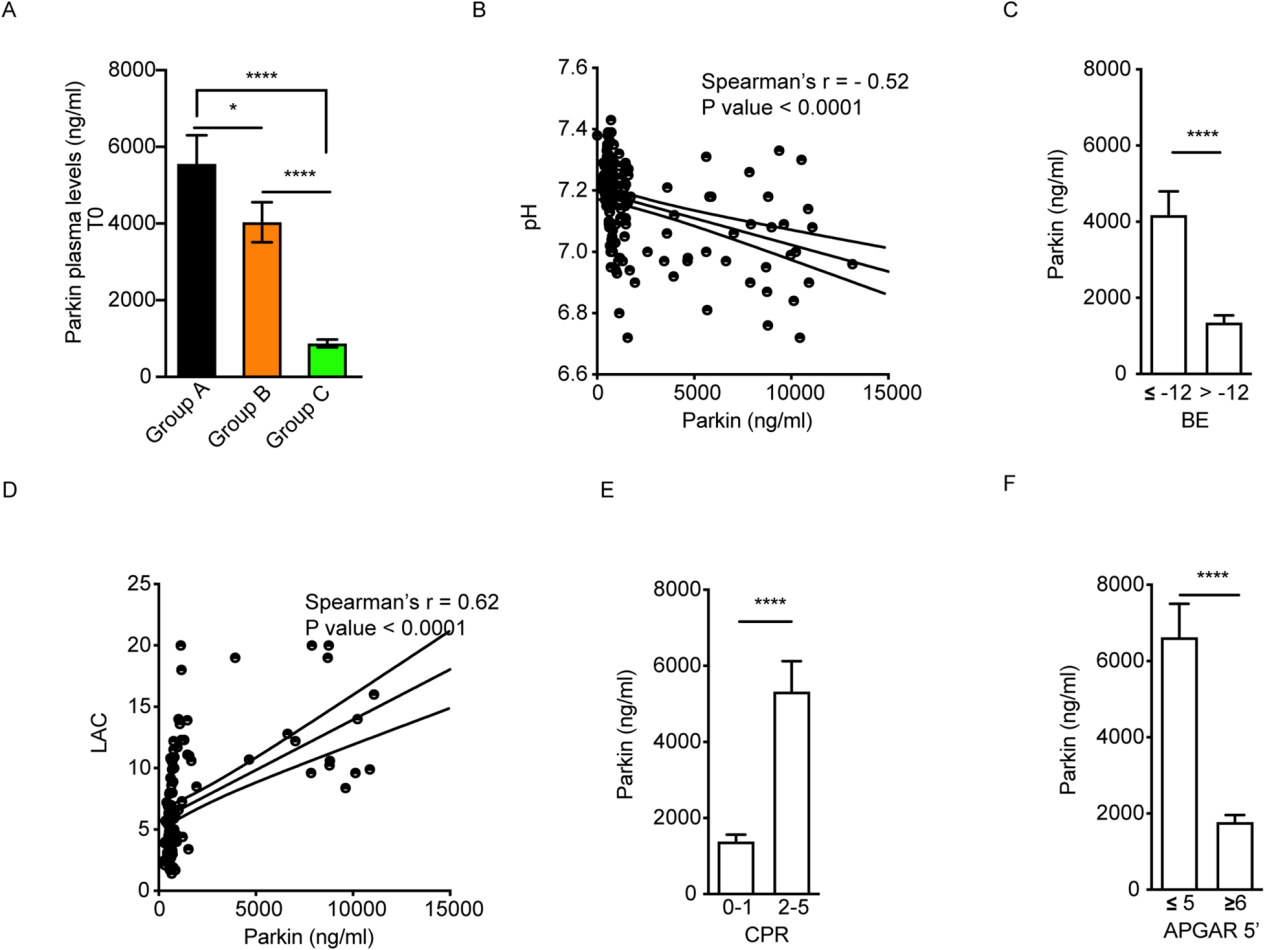
Investigation of mitophagy in newborns at birth and correlation with clinical endpoints. (**A**) Parkin plasma levels measured by ELISA in healthy, babies not needing TH and severe HIE newborns. (**B**) Correlation between Parkin and pH at birth. (**C**) Parkin level stratification according to the base excess score in the whole cohort. (**D**) Correlation between Parkin and lactate release at birth. (**E**) Parkin level stratification according to resuscitation-at- birth score in the whole cohort (0–1 = CPAP assistance, 2–5 = from PPV to drugs). (**F**) Parkin level stratification according to Apgar score at 5 min in the whole cohort.

Plasma concentrations of Parkin were significantly different among the study groups (*P* value < 0.0001). At birth (T0), 4030 ± 522.4 ng/ml circulating Parkin protein was detected in the group B, 5555 ± 744.3 ng/ml in the group A and 869.4 ± 102.9 ng/ml in the group C (Fig. 1A). Newborns with severe HIE (group A) had significantly higher levels of Parkin than those not needing TH (group B) (*P* value < 0.05) and healthy neonates (group C) (*P* value < 0.0001).

From a clinical point of view, at T0, mitophagy was significantly and inversely correlated with the pH of the newborn (Spearman’s r = − 0.52) (Fig. 1B) and directly proportional to lactate release (Spearman’s r = 0.62) (Fig. 1D) in perinatal conditions. Base excess (BE) detected in plasma was also found to be strongly related to mitophagy: lower values of this index were linked to increased circulating Parkin (*P* value < 0.0001) (Fig. 1C). Overall, elevated Parkin levels indicated a worse clinical scenario as shown by the correlation analysis between the circulating protein and Apgar score in which values less than or equal to 5 at 5 min included subjects with significantly higher mitophagy (P value < 0.0001) (Fig. 1F). The highest levels of Parkin (Fig. 1E) were found in almost all newborns who underwent resuscitation at birth (CPR). In conclusion, a high plasma concentration of Parkin correlates with the severity of hypoxic insult at birth.

### Asphyxiated neonates have highest levels of ATG5

**Figure 2:**
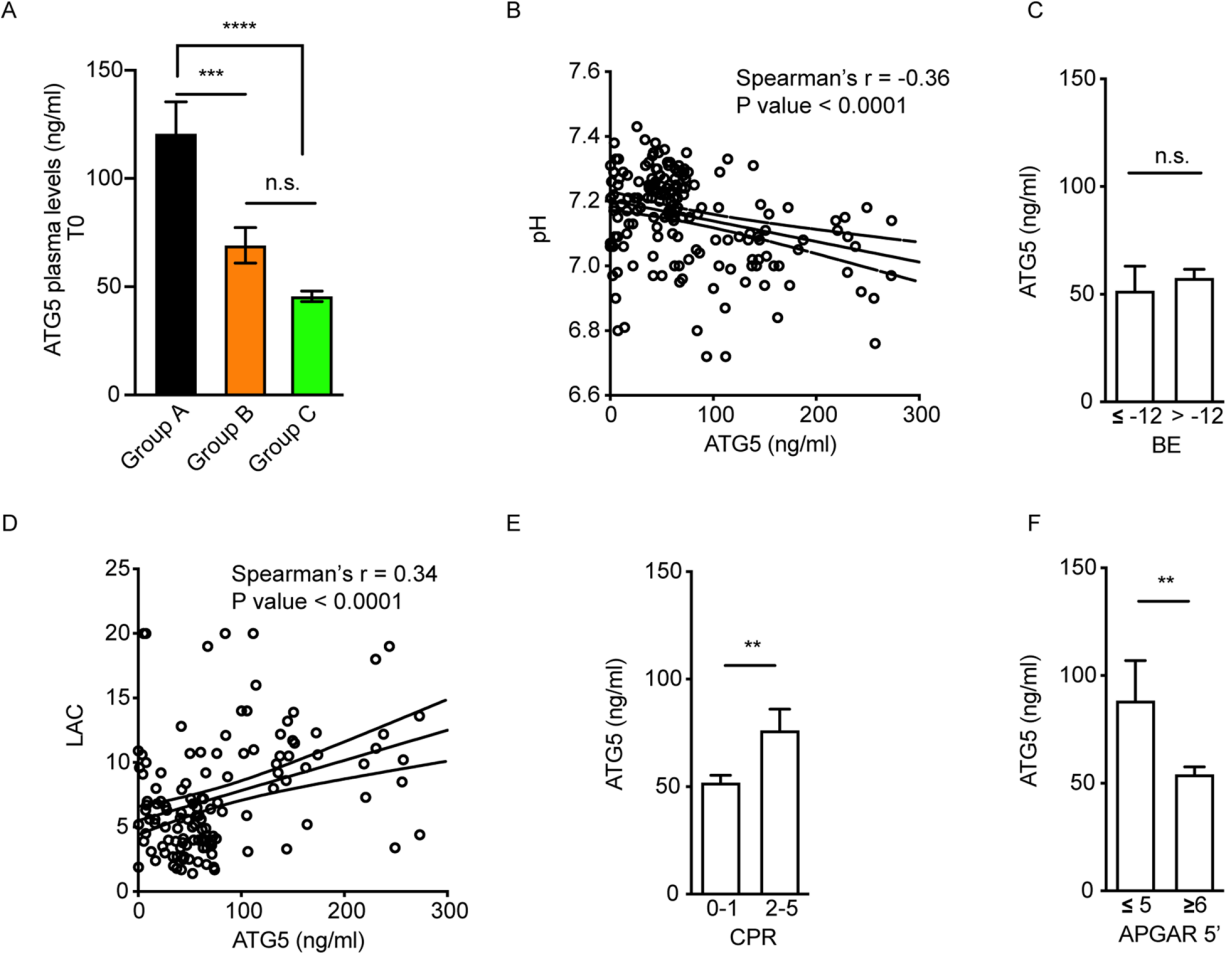
Investigation of autophagy in newborns at birth and correlation with clinical endpoints. (**A**) ATG5 plasma levels measured by ELISA in healthy, babies not needing TH and severe HIE newborns. (**B**) Correlation between ATG5 and pH at birth. (**C**) ATG5 level stratification according to base excess score in the whole cohort. (**D**) Correlation between ATG5 and lactate release at birth. (E) ATG5 level stratification according to resuscitation at birth score in the whole cohort (0–1 = CPAP assistance, 2–5 = from PPV to drugs). (**F**) ATG5 level stratification according to Apgar score at 5 min in the whole cohort.

The increase in autophagy in the blood samples of the same babies was investigated by monitoring the changes in ATG5 release. ATG5 plasma levels were significantly different among the study groups (P value < 0.0001). At T0, 69.13 ± 8.18 ng/ml circulating ATG5 protein was detected in the group B, 120.7 ± 14.78 ng/ml in the group A and 45.59 ± 2.39 ng/ml in the group C (Fig. 2A). Newborns with severe HIE had significantly higher levels of ATG5 than those of the group B not needing TH (*P* value < 0.01) and healthy neonates (*P* value < 0.0001). No differences between healthy newborns and neonates with MAB were detected.

Less but significant correlations were also found for autophagy and metabolic acidosis measured by pH (Spearman’s r = -0.36, R square = 0.12) and lactate release (Spearman’s r = 0.34, R square = 0.14) in newborns (Fig. 2B, D). Accordingly, the involvement of autophagy in the worse clinical outcome of HIE, analyzed in terms of correlation with the Apgar and CPR scores at birth (Fig. 2E, F), was weaker than that of mitophagy and absent for BE index (Fig. 2C).

### Parkin level increase with time in Group B newborns.

**Figure 3:**
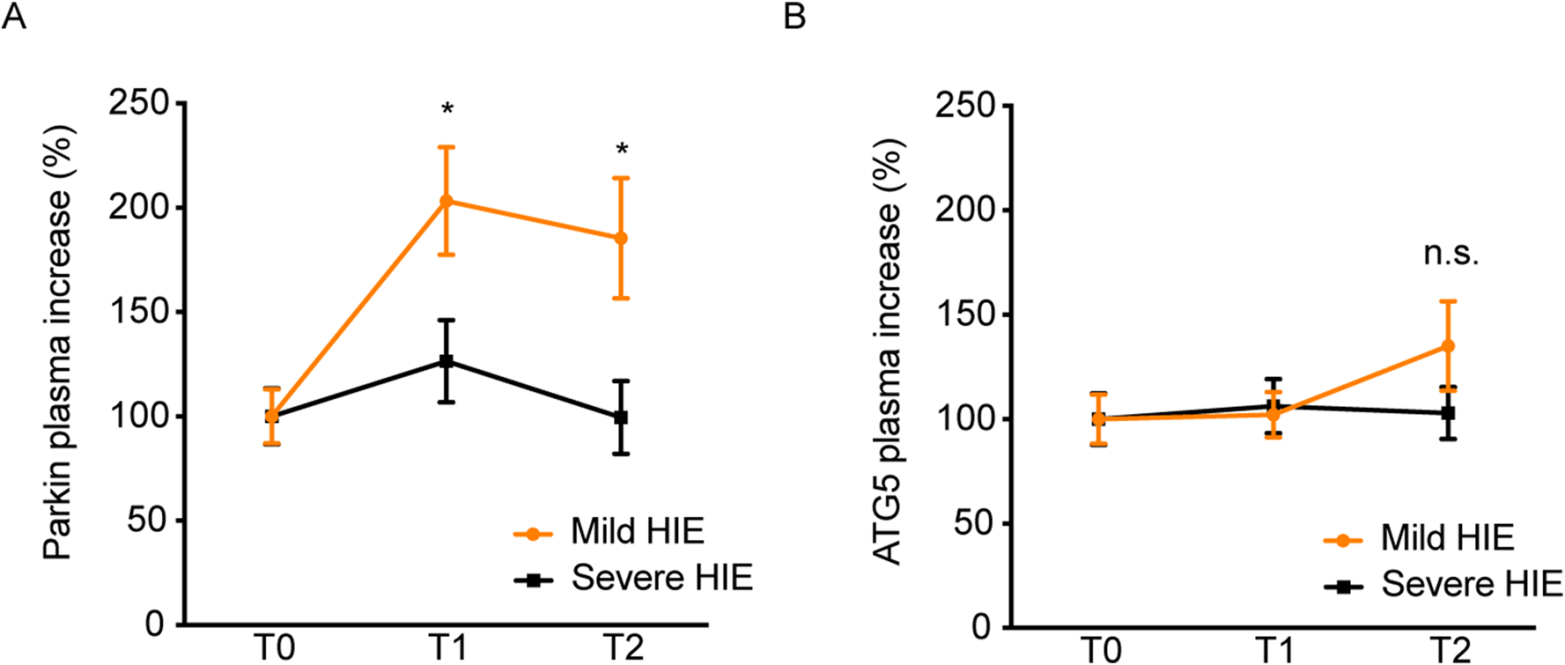
Mitophagy and autophagy changes overtime. (**A**) Parkin plasma levels measured by ELISA in healthy, babies not needing TH and severe HIE newborns at T1 (72 h) and at T2 (7 days), as well as at birth. (**B**) ATG5 plasma levels measured by ELISA in healthy, MAB group and severe HIE newborns at T1 (72 h) and at T2 (7 days), as well as at birth.

By evaluating Parkin values over time (from 0 to 72 h) and setting mean concentrations of the circulating protein to 100% at birth, a significant and constant increase in the group B (metabolic acidosis at birth with or without mild encephalopathy) was recorded (Fig. 3A, *P* value < 0.001). This trend was totally different from that observed for severe HIE treated with TH, which remained unchanged. The increase in circulating Parkin levels in the not hypothermic-cohort (group B) doubled and remained until 7 days. In contrast, in severe HIE, it started decreasing. The biological effect reported for Parkin coincided with the TH of patients affected by severe HIE. In contrast, ATG5 levels did not change between experimental groups and started to increase only at 7 days for B group, but this was not a significant change (Fig. 3B).

### A threshold for Parkin that may be relevant to guide therapeutic strategies in the Group B

**Figure 4:**
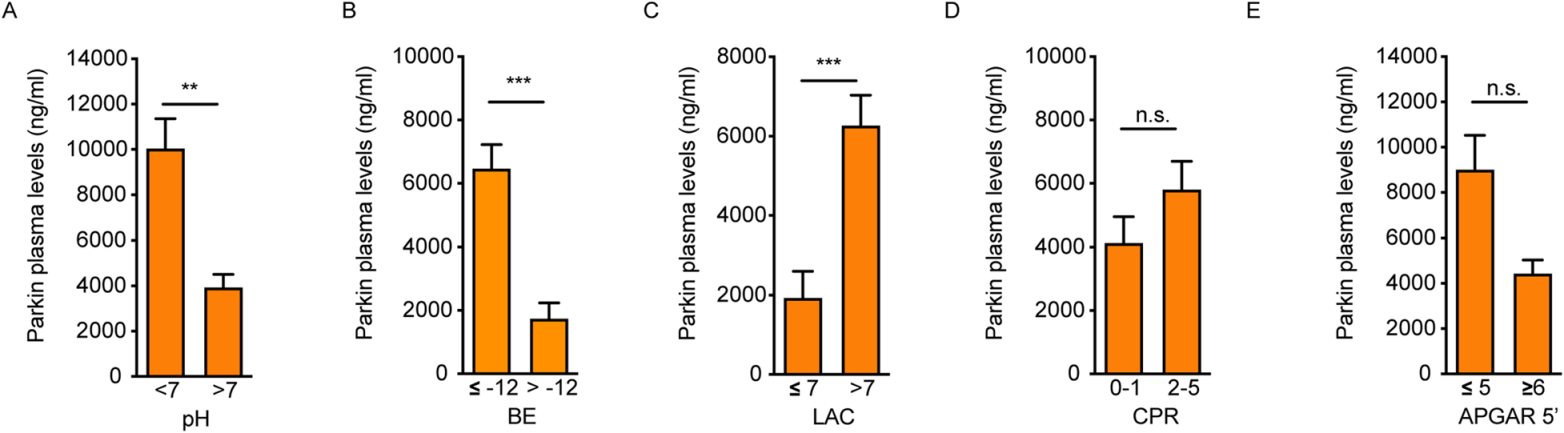
Stratification of Parkin levels in the Group B. Parkin level stratification according to pH (**A**), base excess (**B**), lactates (**C**), CPR (**D**) and Apgar score at 5 min (**E**) in Group B.

Based on data collected in this study, the subsequent analysis was performed to understand if a threshold of circulating Parkin (in ng/ml) at birth exists that would be useful in characterizing either a better or worse clinical scenario. To this end, Parkin levels for the solely not hypothermic-cohort (Group B) were grouped based on pH (< or > 7), BE (≤ or > 12), LAC (≤ or > 7), CPR, and Apgar scores (≤ 5 and ≥ 6) (Fig. 4). Although two of five correlations were not significant, overall, it appeared that Parkin values above the range of 4000 ng/ml in group B might be similar to those of group A.

## Discussion

It is universally accepted that TH is the current standard of care for newborns with encephalopathy thanks to its ability to reduce mortality and morbidity^25^. Despite advances in neonatal intensive care, the evaluation of brain injury risk, particularly in mild HIE, can be complicated.

The diagnosis and prognosis of HIE are based on clinical manifestations, neuroimaging (e.g., head ultrasound and magnetic resonance imaging-MRI) and electrophysiological examinations. However, these tools have some limitations because of the progression of disease factors and the subjectivity of examinations. As a consequence, in the management of infants with neonatal HIE, one of the greatest challenges is in the prediction, detection, and grading of neonatal HIE. Despite the clear guidelines used to identify newborns qualified for cooling, it can be difficult to perform TH because of the complex pathophysiology, uncertain timing, different grades of severity and manifestations.

Currently, the early diagnosis of neonatal HIE depends on observing clinical symptoms and signs using MRI, head ultrasound and EEG.

Despite the high prognostic value of brain MRI, some authors affirmed that normal neuroimages did not consistently equate to normal cognitive and language measures at approximately 24 months of age^26^.

Amplitude-integrated EEG and conventional continuous EEG can detect early changes associated with brain injury with prognostic value as early MRI. aEEG until 6 h of life is the best single outcome predictor in term infants with perinatal asphyxia, but Thoresen et al. affirmed that hypothermia changes the predictive value of early aEEG because normalization of an infant’s aEEG while being cooled occurs later^27^. According to the literature, neurodevelopmental outcomes depend on the severity of the initial insult, and infants with a mild Sarnat score are considered to have a good prognosis without long-term disabilities. For this reason, many studies do not examine the neurological outcome of mild HIE beyond the newborn period, and few randomized controlled trials of TH have been drawn for babies with mild HIE. Emerging evidence suggests that infants with mild HIE who are not treated with TH have an increased risk for both short- and long-term neurodevelopmental impairment^4,28^. Conway et al. showed that newborns affected by mild HIE have a 25% risk of an abnormal neurological outcome, defined as death, cerebral palsy or developmental delay at 18 months^4^. Chalak et al., in the PRIME study, reported that 16% of 43 patients with mild HIE developed neurological disability at the 18–22 month follow-up^24^. The MARBLE prospective multicenter cohort study reported that out of 37 neonates with mild HIE who received TH, one infant developed cerebral palsy, while 20 had mild-to-moderate white matter injury^29^. Last, Goswami published a study showing that, although TH is associated with longer hospitalization and longer durations of respiratory support, mild HIE treated with TH has lower odds of brain injury on imaging than mild HIE not treated with TH^30^. Despite the absence of guidelines, this population is increasingly being offered TH the evidence of its benefit. Infants need to start TH as soon as possible and must be identified rapidly to start TH within 6 h of birth, but HIE is a dynamic brain disorder that evolves over time^31^, and neurological examination can be doubtful to decide for TH. Consequently, a not negligible percentage of infants can be over-treated, and infants that would benefit from TH are not treated.

In this context, the knowledge about specific molecular pathways whose proteins increase within the first hours of life may help in the early diagnosis of HIE to identify neonates qualifying for neuroprotection and improve neonatal survival. Biomarkers are needed to improve management and guide the therapeutic decision. In addition, it will be useful for the evaluation of neonatal HIE therapeutic measures such as mild hypothermia therapy and neuroprotective drugs.

Linked to this, in the last few years, numerous blood biomarkers for the evaluation of perinatal encephalopathy have been studied. Over the past few decades, erythropoietin (EPO), Activin A, NSE, oxidative stress (OS) markers, GFAP, and Creatine Kinase BB (CK-BB) have been investigated^12,32–34^. However, none appears to help in the early identification of babies at risk for neurological impairment to guide neuroprotective strategies.

GFAP, for example, has already been correlated with the damage detected by MRI; however, its variation in the serum is not statistically relevant during the first 6 h of life and cannot be used to guide therapeutic decisions that must be made in the therapeutic window^11,33^. Likewise, NSE, myelin basic protein (MBP), S100B and Tau increase following brain damage^32^, but they have either not been studied at birth or their levels start to be significantly higher only after a few hours. Lactacidemia has also been evaluated as a possible early marker, but further investigations are needed to validate its correlation with the risk of developing encephalopathy. Other markers, such as ubiquitin C-terminal hydrolase-L1 (UCHL-1), rapidly decrease, making the assessment of damage over time impracticable^32^.

In this study, the presence of autophagic and mitophagic circulating proteins were investigated in patients affected by different levels of neonatal brain injury and matched with healthy controls. The results show a strong correlation between HIE grade and the levels of the proteins. Autophagy is a necessary process for cellular survival since it leads to damaged components degradation. Among proteins related to autophagic process, ATG5 has an essential role as it has been shown by the fact that its inhibition leads to the failure of the entire autophagic process^19^.

Mitochondria are organelles that are essential for cellular life and highly susceptible to external insults such as hypoxic events. Therefore, mitophagy is one of the quality control mechanisms that a cell uses to maintain optimal mitochondrial wellbeing. As mitochondrial damage is an early event of hypoxic-ischemic insult, mitophagy was investigated immediately at birth by evaluating the amount of Parkin in the plasma samples of the experimental groups. Parkin is a ubiquitin ligase that, once activated by different factors including hypoxic-ischemic insult, can mark damaged mitochondria addressing them to lysosomal degradation^35^.

What we observed is that patients affected by severe HIE show significantly increased levels of Parkin compared to newborns not needing TH and healthy newborns. The data of this study indicate that the levels of Parkin directly correlate with the severity of damage and progressively increases over time. In addition, the heightened Parkin levels seem to be attenuated with the execution of a therapeutic strategy, such as TH. Thus, patients with severe HIE treated with TH have a lower increase in Parkin levels at T1 and unchanged levels at T2 than patients with metabolic acidosis at birth and/or mild HIE not qualified for TH (Group B).

Regarding autophagy, overall, the differences found among the groups studied remained statistically significant, but the correlation with clinical manifestations was weaker than that with mitophagy. Thus, we considered that dosage to be secondary to Parkin.

In this study, only the plasma levels of these proteins have been evaluated, as their measure in cerebrospinal fluid (CSF) needs an invasive test, which was not feasible for the infants enrolled. Although we understand that the brain origin of these proteins might be doubtful, our previous studies have demonstrated the similar behavior of these autophagic markers in both blood and CSF samples of patients with neurodegenerative disorders^22,23,36^. In addition, ATG5 and Parkin in healthy patients who did not encounter brain damage have the lowest values of the whole population.

The observations made in this study suggest that these proteins are related to the hypoxic-ischemic insult’s severity and, being reliable at the time of birth, they could be also useful to identify the most at-risk in group B, in order to eventually address them to therapeutic strategies. These preliminary observations revealed a threshold of Parkin that could suggest the possible use of the protein as a score to determine the necessity of neuroprotective strategies in mild HIE as well. This conclusion has a predictive basis and needs to be confirmed at the end of the study.

We understand that, at this stage, conclusive statements about the long-term ability of Parkin and ATG5 to correlate with the outcome would be premature and that our results must be taken cautiously, needing further confirmation when the follow-up of all patients will be concluded. Nevertheless, we also believe that the descriptive results of what happens to Parkin and ATG5 concentrations after a hypoxic-ischemic insult should not be underestimated: the fact that following HIE, we found an increase already at T0 of two molecules, which have never been investigated before in this field, represents for itself a breakthrough and deserves further investigations.

### Limitations

The main limitations of this study are the absence of the long-term neurodevelopmental follow-up until 2-years of age and the absence of CSF where the same circulating proteins can be tested.

One minor limitation is the absence of correlation between this study’s markers and the others validated (i.e., MRI scoring) for Group B newborns because no extra procedures were performed for newborns not eligible for TH.

### Study approval

It was approved by the Ethics Committee of the University Hospital of Ferrara and recorded to Clinical Trials as AutophaGy AcIdosis Newborn – AGAIN” (NCT03897101). As reported in the Clinical Trials, all methods were carried out in accordance with Italian guidelines and regulations. The study cohort comprised infants, inborn or outborn with written parental informed consent.

## Data Availability

The authors declare that the data supporting the findings of this study are either available within the paper or are available from the corresponding authors upon reasonable request.
